# Synaptotagmin VII Restricts Fusion Pore Expansion during Lysosomal Exocytosis

**DOI:** 10.1371/journal.pbio.0020233

**Published:** 2004-06-29

**Authors:** Jyoti K Jaiswal, Sabyasachi Chakrabarti, Norma W Andrews, Sanford M Simon

**Affiliations:** **1**Department of Cellular Biophysics, Rockefeller UniversityNew York, New YorkUnited States of America; **2**Section of Microbial Pathogenesis and Department of Cell Biology, Yale University School of MedicineNew Haven, ConnecticutUnited States of America

## Abstract

Synaptotagmin is considered a calcium-dependent trigger for regulated exocytosis. We examined the role of synaptotagmin VII (Syt VII) in the calcium-dependent exocytosis of individual lysosomes in wild-type (*WT*) and Syt VII knockout (*KO*) mouse embryonic fibroblasts (MEFs) using total internal reflection fluorescence microscopy. In *WT* MEFs, most lysosomes only partially released their contents, their membrane proteins did not diffuse into the plasma membrane, and inner diameters of their fusion pores were smaller than 30 nm. In Syt VII *KO* MEFs, not only was lysosomal exocytosis triggered by calcium, but all of these restrictions on fusion were also removed. These observations indicate that Syt VII does not function as the calcium-dependent trigger for lysosomal exocytosis. Instead, it restricts the kinetics and extent of calcium-dependent lysosomal fusion.

## Abstract 2

Lysosomes only partially release their contents in wild-type mouse embryonic fibroblasts (MEFs); but in MEFs in which synaptotagmin VII has been deleted, lysosomes fuse completely

## Introduction

Exocytosis allows cells to transport membrane-impermeable macromolecules outside without compromising the integrity of the plasma membrane. The proteins that form the conserved machinery for constitutive and regulated exocytosis have been identified (Sollner and Rothman 1996), and calcium has been identified as the most common trigger for regulated exocytosis (Burgoyne and Morgan 1998; Jaiswal 2001). However, there is not yet a consensus on the calcium-responsive components involved in this process. It has been suggested that multiple Ca^2+^-binding proteins with distinct properties could act as the trigger for membrane fusion (Burgoyne and Morgan 1998). Evidence supporting the role of synaptotagmin I (Syt I) as the Ca^2+^-dependent trigger for synaptic vesicle fusion in several organisms has led to the belief that the members of the synaptotagmin family act as ubiquitous calcium-dependent triggers for exocytosis (Brose et al. 1992; Geppert et al. 1994; Littleton and Bellen 1995). While Syt I is the most well-studied member of this family, there are at least 15 different synaptotagmin isoforms with differing affinities for calcium and phospholipid and different cellular localization (Chapman 2002; Fukuda 2003). Some members of synaptotagmin family (including Syt I) have also been found to regulate endocytosis and even negatively regulate Ca^2+^-dependent exocytosis (Jorgensen et al. 1995; Martin et al. 1995; Morimoto et al. 1995; Baram et al. 1999; Tucker and Chapman 2002). Thus, the role of synaptotagmin family members as Ca^2+^-dependent triggers for exocytosis is still an open question.

We have previously identified that in nonprofessional secretory cells calcium preferentially triggers exocytosis of lysosomes (Jaiswal et al. 2002). A variety of agents that result in calcium increase, including membrane damage, trypanosome invasion*,* calcium ionophores, or the IP3 agonists thrombin or bombesin, trigger lysosomal exocytosis (Rodriguez et al. 1997; Caler et al. 2000, 2001; Ayala et al. 2001; Reddy et al. 2001; Jaiswal et al. 2002). However, the molecular machinery that regulates this calcium-triggered lysosomal exocytosis has remained elusive. Syt VII is the synaptotagmin isoform present on lysosomes (Martinez et al. 2000). It is expressed in most tissues and is present in organisms ranging from nematodes to humans (Fukuda et al. 2002). Syt VII is involved in processes requiring lysosomal exocytosis, namely, release of lysosomal enzymes, repair of membrane rupture, and trypanosome invasion (Martinez et al. 2000; Caler et al. 2001; Reddy et al. 2001). Further, the recent demonstration that cells from Syt VII knockout (*KO*) mice are compromised in these functions supports a role of Syt VII in regulating lysosomal exocytosis (Chakrabarti et al. 2003). To understand how Syt VII regulates lysosomal fusion, we used total internal reflection fluorescence microscopy (TIR-FM) and studied the behavior of individual lysosomes following calcium increase in mouse embryonic fibroblasts (MEFs) from wild-type (*WT*) and Syt VII *KO* mice.

## Results

**Figure 1:**
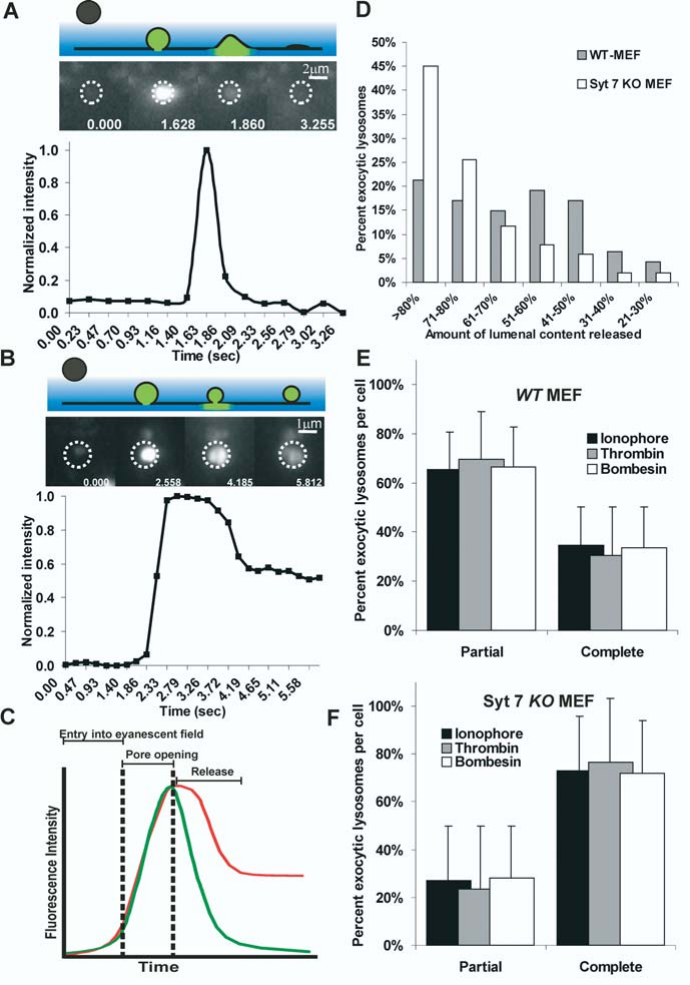
Fate of Lumenal Content during Lysosomal Fusion MEFs were incubated for 2 h with 70 kDa FITC–dextran followed by more than 3 h in dextran-free media to chase the dextrans into the lysosomes. These cells were then treated with calcium ionophore (A23187) to trigger exocytosis of lysosomes. (A and B) The middle panels are images of lyosomes undergoing complete (A) and partial (B) exocytosis. Intensity plots for the regions in these images marked by dotted circles are shown in the lower panel. The top panel shows a schematic representation of these different stages. (C) Schematic fluorescence intensity plots for lysosomes undergoing partial (red) or complete (green) fusion. Owing to the exponential decay of the evanescent field (blue; top panels in [A] and [B]) away from the coverslip, a lysosome that is more than 150 nm from the cell membrane (black line in top panels in [A] and [B]) is not fluorescent. As this lysosome moves closer (labeled as “entry into evanescent field”), its fluorescence intensity increases. Since the lumen of lysosome is acidic, it quenches FITC fluorescence. As soon as the fusion pore is formed, the lysosomal lumen is rapidly alkalinized resulting in an increase of FITC–dextran fluorescence (“pore opening”). Following the pore opening, the dextran is released and it diffuses away from the site of the fusion, causing the lumenal fluorescence to decrease (“release”). (D) A histogram of the fraction of lumenal contents released by exocytosing lysosomes. Upon ionophore-triggered fusion, 21% of all lysosomes analyzed in *WT* MEFs (*n* = 47; gray bars) and 45% of all in Syt VII *KO* MEFs (*n* = 51; white bars) completely released their lumenal content. (E and F) To monitor the nature of lysosomal fusion in individual *WT* MEFs (E) and Syt VII *KO* MEFs (F), calcium was increased using ionophore (*WT*, *n* = 7 cells; *KO*, *n* = 9 cells) as well as the IP3 agonists bombesin (*WT*, *n* = 6 cells; *KO*, *n* = 9 cells) and thrombin (*WT*, *n* = 5 cells; *KO*, *n* = 7 cells). Irrespective of the means, increase in calcium led to most lysosomes to fuse partially in *WT* MEFs (E) and completely in Syt VII *KO* MEFs (F). The error bars represent SEM.

To monitor the fate of exocytic lysosomes in MEFs, we labeled their lumen using fluorescent dextran (FITC–dextran). Treating MEFs with calcium ionophore A23187 or the IP3 agonist bombesin or thrombin caused lysosomal exocytosis (Figure 1A and 1B). Fusion of a FITC–dextran-loaded lysosome was indicated by a transient increase followed by a decrease in its fluorescence (Figure 1A–1C). The increase in fluorescence was due to a combination of two factors: (a) movement of the lysosome closer to the coverslip, which results in better excitation of its cargo by the evanescent wave; (b) opening of the fusion pore, which results in dissipation of the acidic pH of the lysosomes, resulting in dequenching of the fluorescence of FITC–dextran. The rapid decrease in fluorescence was due to the diffusion of lumenal cargo away from the site of fusion (Figure 1A–1C). In some of the exocytosing lysosomes, the lumenal fluorescence decreased down to baseline, indicating that they completely released their lumenal cargo (Figure 1A). The fluorescence of other lysosomes did not decrease down to baseline at the site of fusion (Figure 1B). Thus, these lysosomes only partially released their contents upon fusion. To resolve whether partial release represented a very slow diffusion of lumenal content or an opening of the fusion pore that was transient, we observed the lysosomes for longer periods. During partial release, the lumenal fluorescence decreased rapidly within the first second (Figure 1B), but remained relatively constant afterwards, decreasing only at the rate of photobleaching (*t*_1/2_ for FITC in our setup is 28.5 s). Absence of any subsequent decrease in its fluorescence, even over longer periods, indicated that cessation of release was the result of closure of the fusion pore prior to complete release of the lumenal cargo. Quantitation of the lumenal contents retained in all exocytosed lysosomes analyzed in the *WT* MEFs revealed that only 21% completely released their lumenal content (Figure 1D, gray bar). The percentage of lysosomes in individual *WT* MEFs that only partially released their lumenal cargo of 70 kDa dextran in response to A23187-induced increase in cellular Ca^2+^ was 65.3% (*n* = 7 cells) (Figure 1E, black bars; Table 1; Video S1). A comparable fraction of lysosomes, respectively, underwent partial release when calcium increase was triggered by the IP3 agonists thrombin (66.3) or bombesin (69.5), (Figure 1E; Table 1).

**Table 1:**
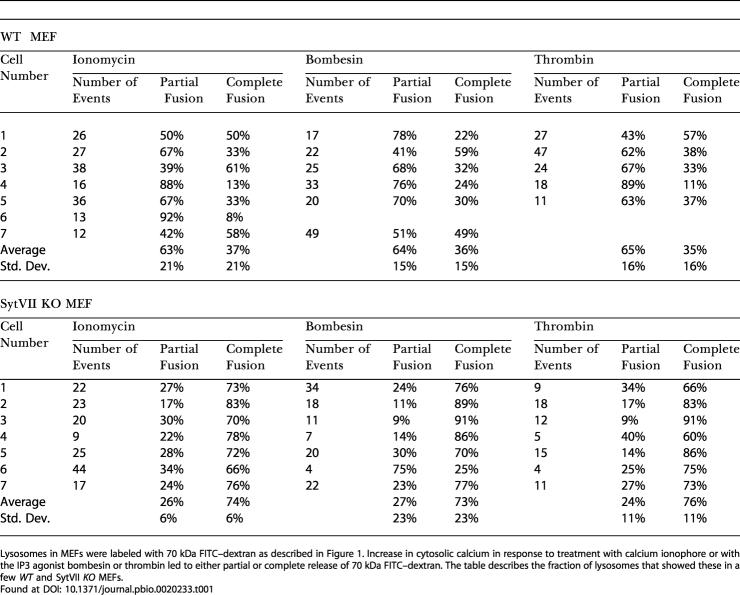
Nature of Release of Lysosomal Lumenal Content

Lysosomes in MEFs were labeled with 70 kDa FITC–dextran as described in Figure 1. Increase in cytosolic calcium in response to treatment with calcium ionophore or with the IP3 agonist bombesin or thrombin led to either partial or complete release of 70 kDa FITC–dextran. The table describes the fraction of lysosomes that showed these in a few *WT* and SytVII *KO* MEFs

Found at

**Figure 2:**
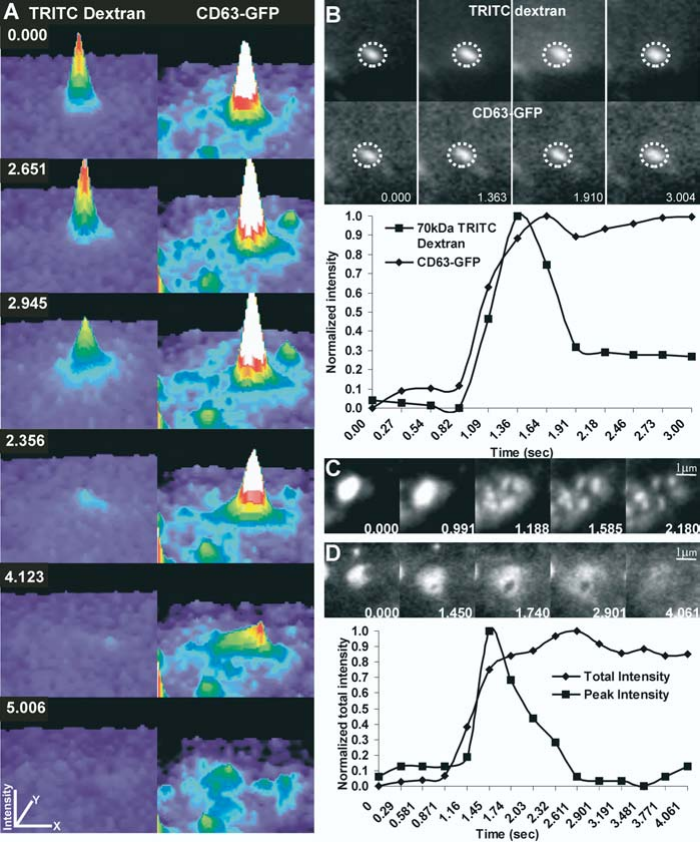
Fate of Membrane Protein during Lysosomal Fusion Lysosomal membranes in MEFs were labeled by transfecting cells with a vector encoding a CD63–GFP fusion protein, and expression was allowed for 48 h. For simultaneous labeling of lysosomal membrane and lumen, the CD63–GFP transfected cells were labeled with 70 kDa TRITC–dextran as described in Figure 1. (A) Following ionophore-induced calcium increase in *WT* MEFs, when the TRITC–dextran was released completely (left), CD63–GFP (right) was delivered to the plasma membrane, but it remained in multiple puncta near the site of fusion rather than diffuse away. The panels are pseudocolor surface plots, with the *x* and *y* axis representing the coordinates and the *z* axis representing the fluorescence intensity of individual pixels. (B) In the event of partial release of TRITC–dextran (top row), the CD63–GFP (bottom row) did not appear to be delivered to the plasma membrane. The lower panel shows the plot of fluorescent intensity of lumenal and membrane label (within the dotted circle) of the lysosome shown in (B). (C and D) Analysis of CD63–GFP-labeled lysosomes in *WT* MEFs (C) and in Syt VII *KO* MEFs (D) indicates that while CD63–GFP is retained in puncta in the *WT* MEFs, it diffuses freely in the plasma membrane in the SytVII *KO* MEFs. The lower panel shows the total and peak intensity plots of CD63–GFP-labeled lysosome in (D).

In order to determine the fate of the membrane proteins during Ca^2+^-triggered lysosomal exocytosis, we simultaneously imaged the lysosomal lumenal (TRITC–dextran) and membrane (CD63–GFP) cargoes in *WT* MEFs. We observed that the membrane proteins were delivered to the plasma membrane during complete release of lumenal content (Figure 2A), but not when the lumenal contents were released partially (Figure 2B). Even when the lysosomal membrane proteins were delivered to the plasma membrane, their diffusion into plasma membrane was restricted (Figure 2A and 2C; Table 2). This is unlike the fate of membrane proteins during exocytosis of biosynthetic vesicles (Schmoranzer et al. 2000; Kreitzer et al. 2003; Schmoranzer and Simon 2003) or recycling endosomes (Lampson et al. 2001), where following its delivery to plasma membrane, the vesicular membrane protein diffuses away completely from the site of fusion. Thus, exocytosis of lysosomes in the *WT* MEF is different from other exocytic events in two manners. First, the majority of exocytic lysosomes only partially release their lumenal cargo with no release of membrane proteins. Second, even when the lumenal cargo is completely released, the membrane proteins delivered to the plasma membrane do not diffuse freely, but are retained into punctae at the site of fusion.

**Table 2:**
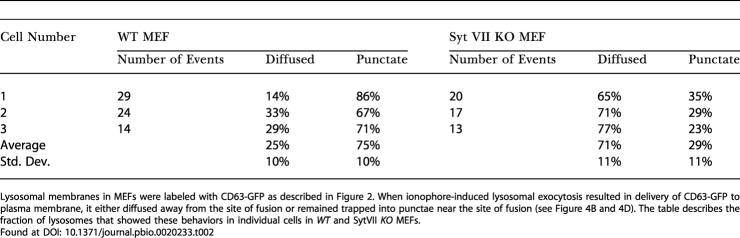
Nature of Release of Lysosomal Membrane Protein

Lysosomal membranes in MEFs were labeled with CD63-GFP as described in Figure 2. When ionophore-induced lysosomal exocytosis resulted in delivery of CD63-GFP to plasma membrane, it either diffused away from the site of fusion or remained trapped into punctae near the site of fusion (see Figure 4B and 4D). The table describes the fraction of lysosomes that showed these behaviors in individual cells in *WT* and SytVII *KO* MEFs

Found at

To test the role of Syt VII in regulating these processes and acting as a calcium-dependent trigger for lysosomal exocytosis, we carried out a similar analysis using embryonic fibroblasts from mice deficient in Syt VII (Syt VII *KO* MEF) (Chakrabarti et al. 2003). Absence of Syt VII did not abolish calcium-dependent triggering of lysosomal fusion in response to ionophore, thrombin, or bombesin (see Figure 1D and 1F; Video S2). Upon examining individual exocytic lysosomes, we observed two significant differences between the *WT* and Syt VII *KO* MEFs. First, in individual Syt VII *KO* MEFs, a significantly greater (2-fold) fraction of lysosomes completely released their lumenal contents when the cellular calcium was raised using ionophore (*p* = 0.001) (Video S2), thrombin (*p* = 0.001), or bombesin (*p* = 0.003) (see Figure 1F; see Table 1). Second, upon complete fusion, the membrane protein of most exocytic lysosomes in Syt VII *KO* MEFs diffused freely in the plane of plasma membrane (see Figure 2D; see Table 2). These phenotypes indicated that the presence of Syt VII restricts complete fusion of lysosomes.

**Figure 3:**
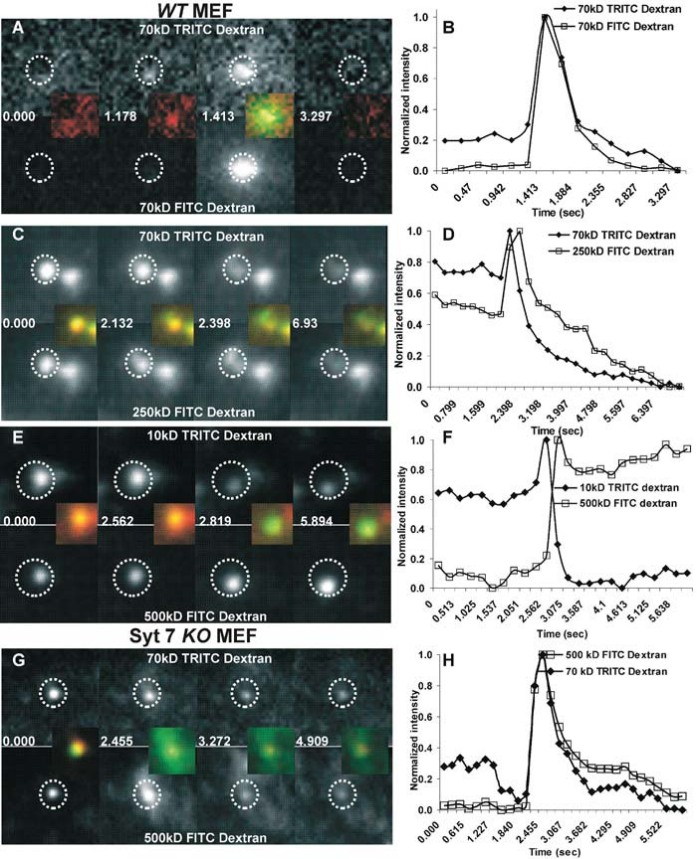
Presence of Syt VII Restricts the Size of the Fusion Pore The lumen of lysosomes was loaded simultaneously using different-sized FITC- and TRITC-labeled dextran, using the approach described in Figures 1 and 2. Representative plots shown here demonstrate the fate of both dextran populations in individual lysosomes following the increase in intracellular calcium by addition of calcium ionophore. In *WT* MEFs, exocytosing lysosomes that released 70 kDa TRITC–dextran also simultaneously released 70 kDa FITC–dextran (A and B), 250 kDa FITC–dextran (C and D), but not 500 kDa FITC–dextran (E and F). In Syt VII *KO* MEFs, lysosomes that released 70 kDa TRITC–dextran also released 500 kDa FITC–dextran (G and H). In all cases the plots represent the normalized fluorescence intensity of the region marked in the images by dotted circles.

To identify whether Syt VII restricts lysosomal fusion by preventing its flattening into the plasma membrane or by regulating the size of the fusion pore, we quantified the simultaneous release of dextrans of different sizes in individual lysosomes (10 kDa [Stokes radius = 2.4 nm], 70 kDa [5.8 nm], 145 kDa [8 nm], 250 kDa [10.5 nm], and 500 kDa [14.7 nm]). Lysosomes were loaded with dextrans of two different sizes, each tagged with a different fluorophore (FITC or TRITC). In *WT* MEFs, all the lysosomes that released the TRITC–dextran of 10 or 70 kDa also released similarly sized FITC–dextran (Figure 3A and 3B). Thus, the fluorophore did not appear to affect release of the dextran. The fluorophore also had no affect on the lysosomal uptake of the dextran, as every lysosome that had the TRITC-labeled dextran also had the FITC-labeled dextran (data not shown). Further, just prior to fusion, the fluorescence of the TRITC cargo and the FITC cargo in each lysosome started to increase at the same moment (Figure 3). This increase is the result of the movement of the lysosome to the plasma membrane just prior to fusion, which increases the excitation of the fluorophores (see Figure 1A–1C). Thus, the TRITC and FITC cargos were not only spatially and temporally coincident in the plane of the plasma membrane, but also coincident in the plane perpendicular to the plasma membrane even in a motile lysosome. These observations rule out the possibility that the two fluorophores are present in separate lysosomes.

**Figure 4:**
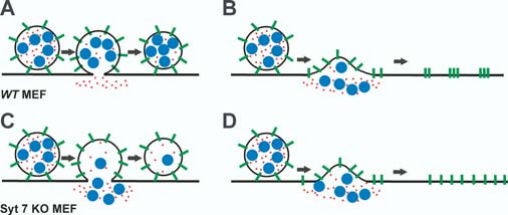
Schematic Representation of the Fate of Lumenal and Membrane Cargo during Lysosomal Exocytosis (A) Partial fusion of the lysosomes from *WT* MEFs result in the release of a fraction of the smaller (70–250 kDa; red circles) dextran, but no release of the larger (500 kDa; blue circles) dextran. None of the lysosomal membrane protein (green bars) is delivered to the plasma membrane. (B) Complete fusion leads to release of both the large and the small dextrans and delivery of the membrane proteins to the plasma membrane, but the proteins aggregate in small puncta near the site of fusion. (C) Knockout of Syt VII causes larger-sized dextran to be released even during partial fusion, and the membrane protein is still not delivered to the plasma membrane. (D) During complete fusion, both sized dextrans are released completely and the membrane proteins delivered to plasma membrane are free to diffuse away from the site of fusion.

In *WT* MEFs, each lysosome that partially released the 10 or 70 kDa TRITC–dextran at the same time also partially released the 145 kDa (data not shown) and 250 kDa FITC–dextran (Figure 3C and 3D). However, when the lysosome was simultaneously loaded with 500 kDa FITC–dextran and with 70 kDa TRITC–dextran, the smaller cargo was released and the larger cargo was not (Figure 3E and 3F). This suggests the fusion pore opened large enough to release the smaller but not the larger cargo (Figure 4). Furthermore, at the moment of release of the TRITC cargo, the fluorescence from the 500 kDa FITC–dextran increased to a significantly greater amount. As the FITC fluorescence is partially quenched by the acidic lumen of the lysosomes, this provided additional evidence in favor of the conclusion that the lysosome formed a fusion pore, without releasing the 500 kDa dextran. The lack of subsequent decrease in the fluorescence of the 500 kDa FITC–dextran, when the 70 kDa TRITC–dextran fluorescence decreases, indicates that the radius of the fusion pore is smaller than the size of the 500 kDa dextran (Stokes radius = 14.7 nm; diameter, 29.4 nm). We repeated these studies in Syt VII *KO* MEFs and found that in these cells all exocytosing lysosomes were able to fully release dextrans of all sizes, including the 500 kDa dextran (see Figure 3G and 3H). This suggests that the presence of Syt VII blocks complete release of lysosomal contents during exocytosis, potentially by restricting the size of the exocytic pore.

**Figure 5:**
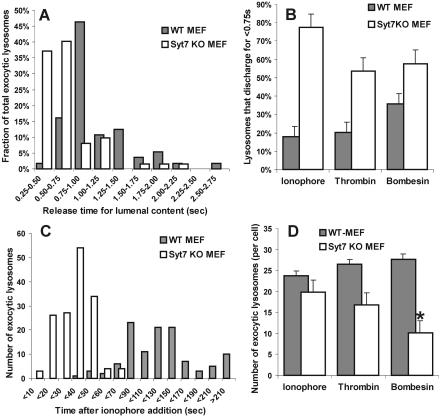
Temporal Analysis of Lysosomal Exocytosis and Fusion Pore Opening Using 70 kDa FITC–Dextran as Lumenal Marker (A) Histogram of the release time (time taken for the vesicular fluorescence to drop from peak to postfusion resting value). Release time was less than 0.75 s for more than two-thirds of lysosomes in Syt VII *KO* MEF (*n* = 62 lysosomes), while most lysosomes (81%) in *WT* MEFs (*n* = 56 lysosomes) released their lumenal content for more than 0.75 s. (B) Analysis of release time of lysosomes using ionophore, bombesin, and thrombin to trigger lysosomal exocytosis. Irrespective of the means of calcium increase, lysosomes in Syt VII *KO* MEFs released their lumenal content significantly faster (*p* = 0.002, 0.01, and 0.03, respectively). (C) Histogram of the number of lysosomes exocytosing as a function of the time following calcium ionophore addition. Fluorescent dextran was used as a lumenal marker; the time axis indicates seconds elapsed following the addition of ionophore. Exocytosis initiated earlier in the Syt VII *KO* MEFs (white bars; *n* = 8 cells) compared to *WT* MEFs (gray bars; *n* = 6 cells). (D) No change was observed in the total number of lysosomes that exocytosed at the basal surface of *WT* or Syt VII *KO* MEFs when calcium was raised using ionophore or thrombin; however, compared to *WT* MEFs, bombesin triggered exocytosis of half as many lysosomes in the Syt VII *KO* MEFs (asterix represents *p* value < 0.02). The error bars represent SEM.

Since cargo of any size would be released more rapidly through a larger pore, we independently assayed for the size of the fusion pore by measuring the time taken by exocytosing lysosomes to release their lumenal cargo. Increase in FITC–dextran fluorescence was taken as the indicator for the time of opening of the fusion pore (see Figure 1C). We measured the time taken for the lumenal fluorescence of individual exocytic lysosomes (fusing partially or completely) to decrease to the post-fusion resting value in *WT* and Syt VII *KO* MEF (see Figure 1C). For most lysosomes (greater than 81%) in *WT* MEFs, it took longer than 0.75 s for the fluorescence of the lumenal cargo to reach their post-fusion resting value (gray bars in Figure 5A). In contrast, in Syt VII *KO* MEFs for most lysosomes (greater than 75%), this occurred in less than 0.75 s (white bars in Figure 5A). The increased propensity of lysosomes to rapidly release their lumenal content was also observed when Syt VII *KO* cells were treated with bombesin or thrombin (Figure 5B). This suggests that in the absence of Syt VII, the fusion pore either opens faster, opens to a larger size, or both. While we cannot distinguish among these possibilities, they are all consistent with Syt VII restricting the expansion of the fusion pore.

To further explore potential consequences of the presence of Syt VII on lysosomal secretion, we analyzed the effect of Syt VII knockout on the time course of initiation of lysosomal exocytosis. Similar to what we observed previously with CHO cells (Jaiswal et al. 2002), in *WT* MEFs, lysosomal exocytosis was initiated approximately 35 s after the addition of ionophore and peaked by 110 s (see Video S1; gray bars in Figure 5C). In contrast, in Syt VII *KO* MEFs, the earliest lysosomal fusion event was observed within 7 s after the addition of ionophore, and it peaked within 40 s (see Video S2; white bars in Figure 5C). As the time delay for IP3 agonist-induced calcium release varied significantly among the cells within a dish, we could not determine whether these agents have a similar affect on the latency of calcium-triggered lysosomal fusion in Syt VII *KO* MEFs.

While all the behaviors of individual lysosomes described above were independent of the agent used to trigger calcium increase, the bulk cellular behavior, such as the total number of lysosomal fusion events that occur at the basal surface of the cell, was dependent on the agent used to increase the cellular calcium level (Figure 5D). While ionophore or thrombin triggered exocytosis of a similar number of lysosomes (*p* = 0.46 and 0.51, respectively) in both *WT* and *KO* MEFs, there was a 2-fold decrease (*p* = 0.02) when bombesin was used to raise cellular calcium in *KO* MEFs.

## Discussion

It has been shown that biosynthetic vesicles, secretory granules, and synaptic vesicles can undergo partial release (Holroyd et al. 2002; Aravanis et al. 2003; Gandhi and Stevens 2003; Schmoranzer and Simon 2003; Taraska et al. 2003). However, our analysis of calcium-triggered fusion of individual lysosomes has revealed several unique features of this process. In *WT* MEFs, partial fusion is the predominant mode of lysosomal exocytosis. Unlike partial fusion of secretory granules in PC12 cells, which results in incomplete release of the large lumenal cargo (Holroyd et al. 2002; Taraska et al. 2003), partially exocytosing lysosomes do not release any of the large (500 kDa) lumenal cargo. Similarly, during partial release of lumenal contents, none of the lysosomal membrane protein diffuses into the plasma membrane. The complete release of lysosomal cargo is also unlike the complete release of biosynthetic vesicles, where the membrane proteins of secretory vesicles fully diffuse into the plasma membrane (Schmoranzer and Simon 2003). In constrast, the lysosomal membrane proteins remain as puncta near the site of fusion. These unique features associated with lysosomal exocytosis are dependent on the presence of Syt VII. Absence of Syt VII causes most lysosomes to fuse completely and allows the membrane proteins of completely fusing lysosomes to diffuse freely into the plasma membrane. Moreover, in the presence of a functional Syt VII, opening of the fusion pore is restricted both temporally (the pores open more slowly and remain open for a short period of time) and geometrically (large lumenal cargo cannot leave and membrane proteins cannot diffuse out into the plasma membrane). Thus, our analysis reveals that Syt VII is not critical for calcium-dependent triggering of lysosomal exocytosis. This is consistent with what has been proposed earlier and observed both in vitro and in vivo for Syt I (Popov and Poo 1993; Martin et al. 1995; Morimoto et al. 1995; Mahal et al. 2002). The effects of Syt VII on the kinetics, size, and extent of calcium-triggered exocytosis of lysosomes in *WT* MEF are also consistent with observations reported for Syt I: the slower dilation of fusion pores caused by overexpression of Syt I (Wang et al. 2001), and a 10-fold increased frequency of asynchronous release in Syt I null cells (Yoshihara and Littleton 2002). Interestingly, unlike the knockout of Syt I, which blocks the fast component of calcium-triggered exocytosis in neuronal cells (Yoshihara and Littleton 2002), knockout of Syt VII did not abolish calcium-triggered exocytosis in the MEFs. This could reflect different roles played by Syt VII (inhibitor for lysosome fusion) and Syt I (calcium-dependent trigger) in calcium-dependent exocytosis. Alternatively, each synaptotagmin may play multiple roles, including being a trigger (Geppert et al. 1994), an inhibitor of asynchronous release (Yoshihara and Littleton 2002), or an inhibitor of fusion pore dilation (Wang et al. 2001). While it remains to be determined whether other members of the synaptotagmin family can act as inhibitors of fusion, this possibility is supported by the recent observation that overexpression of Syt IV causes increased partial release of secretory granules in PC12 cells (Wang et al. 2003). Involvement of Syt VII in premature closure of the fusion pore (leading to partial release) is also consistent with the proposed role of Syt I in facilitating the rapid retrieval of vesicular membrane following exocytosis (Jorgensen et al. 1995).

Syt VII function has been shown to be crucial for membrane repair and trypanosome invasion (Martinez et al. 2000; Caler et al. 2001; Chakrabarti et al. 2003). Cells lacking a functional Syt VII show reduced membrane repair and Trypanosoma cruzi invasion (Chakrabarti et al. 2003). Our observations suggest a few mechanisms by which Syt VII may contribute. Syt VII *KO* MEFs lose the restricted fusion of lysosomes: unlike the lysosomes of *WT* MEFs, the membrane-proximal lysosomes in Syt VII *KO* MEFs fully release their contents and deliver their membrane proteins to the surface. Since membrane of lysosomal origin has been shown to be required for healing membrane rupture and forming parasitophorous vacuoles during trypanosome invasion, it is possible that retaining the lysosomal membrane components at the site of fusion aids in both these processes. Additionally, upon treatment with bombesin, which may recapitulate the calcium signaling occurring during T. cruzi invasion (Tardieux et al. 1994), total lysosomal exocytosis is decreased 2-fold in the Syt VII *KO* MEF. This effect is reminiscent of the approximately 2-fold decrease in lysosomal enzyme secretion observed by collagen matrix-mediated wounding of Syt VII *KO* MEFs (Chakrabarti et al. 2003). We have previously observed that calcium predominantly triggers the exocytosis of membrane-proximal lysosomes (Jaiswal et al. 2002). In the Syt VII *KO* MEFs, these lysosomes also showed decreased propensity for partial release. Thus, it is possible that the membrane-proximal lysosomes play a role in decreased membrane repair and trypanosome invasion observed in the Syt VII *KO* MEFs (Chakrabarti et al. 2003). Our analysis of Syt VII function not only adds to the roles of synaptotagmin in regulating calcium-triggered exocytosis, but also provides mechanistic clues regarding how lysosomal exocytosis might regulate membrane repair and pathogen invasion.

## Materials and Methods

### 

#### Cell growth and treatments

MEFs were prepared from day 13.5 embryos of *WT* and Syt VII-deficient mice, expressing functional (*WT* MEF) or truncated Syt VII (*KO* MEF), as described elsewhere (Chakrabarti et al. 2003). Cells were cultured in DMEM (Cellgro, Mediatech, Washington, District of Columbia, United States) supplemented with 10% FBS (GIBCO Technologies, Carlsbad, California, United States). All experiments were done with cells between passages 1 and 3. For imaging, cells were plated for more than 24 h on sterile glass coverslips (Fisher Scientific, Hampton, New Hampshire, United States). Just before imaging, the medium was replaced with cell imaging medium (CIM) (HBSS plus 10 mM HEPES plus 1% FBS [pH 7.4]). Transient transfection of cells with CD63–GFP (Blott et al. 2001) was carried out using Lipofectamine 2000 (Invitrogen Corporation, Carlsbad, California, United States) 48 h prior to imaging. For calcium ionophore and calcium agonist treatments, growth medium was replaced with CIM, the coverslip was mounted on the microscope stage maintained at 37 °C, and while the cells were being imaged using TIR-FM, agents were added to a final concentration of 10 μM A23187 ionophore, 0.2 U/ml thrombin, or 20 nM bombesin. Calcium ionophore-A23187, thrombin, bombesin, 70 kDa FITC–dextran, and 65 kDa TRITC–dextran were obtained from Sigma (Sigma Chemicals, St. Louis, Missouri, United States). All other fluorescent dextrans were obtained from Molecular Probes (Eugene, Oregon, United States) and used to load lysosomes as previously described (Jaiswal et al. 2002).

#### TIR-FM

The illumination and image acquisition using TIR-FM was done as previously described (Jaiswal et al. 2002). For simultaneous dual-color imaging of GFP/TRITC or FITC/TRITC, we used an emission splitter (Dual-View, Optical Insights, Santa Fe, New Mexico, United States). Cells were excited using the 488 nm line of an argon laser, containing the emission band pass filters (GFP/FITC, HQ525/50M; TRITC, HQ580LP). All filters were obtained from Chroma Technologies Corporation (Brattleboro, Vermont, United States). Images were acquired with a 12-bit cooled CCD ORCA-ER (Hamamatsu Photonics, Hamamatsu, Japan) with a resolution of 1280 × 1024 pixels (pixel size = (6.45 μm)^2^). The camera and mechanical shutters (Uniblitz, Vincent Associates, Rochester, New York, United States) were controlled using MetaMorph (Universal Imaging, Downingtown, Pennsylvania, United States). Images were acquired at 5–10 frames/s. Images containing a region of interest of the cell were streamed to memory on a PC during acquisition and then saved to hard disk. The depth of the evanescent filed for the Apo 60× N.A. 1.45 lens (Olympus Scientific, Melville, New York, United States) was typically approximately 70–120 nm (Schmoranzer et al. 2000).

#### Image processing and quantitative analysis

For dual-color video sequences, the images acquired through the emission splitter were separated, subtracted for background fluorescence, aligned within accuracy of a single pixel, and analyzed using MetaMorph. For measuring fluorescence intensity, a region was drawn around the vesicle and the peak and average intensity was measured in this region. The minimum and maximum average intensities were normalized on a scale of 0 to 1. For measurement of total number of exocytic lysosomes in a cell, fusion events were counted starting from the addition of ionophore until the cells start to lift off the coverslip (1–3 min in *WT* and fewer than 2 min in Syt VII *KO* MEFs).

## Supporting Information

Video S1 Lysosomal exocytosis in *WT* MEFs Lysosomes of *WT* MEFs were loaded with 70 kDa FITC–dextran and the cells were treated with 10 μM calcium ionophore. The video shows a single cell 30 s after ionophore addition. The images were acquired at five frames per second, and alternate frames are displayed in the video at five frames per second. The time stamp indicates the time (mm:ss) elapsed since the start of the video.
(6.8 MB AVI).

Video S2 Lysosomal exocytosis in Syt VII *KO* MEFs Lysosomes in Syt VII *KO* MEFs were loaded with 70 kDa FITC–dextran and the cells were treated with 10 μM calcium ionophore. The video shows a single cell immediately following ionophore addition. The images were acquired at five frames per second, and alternate frames are displayed in the video at five frames per second. The time stamp indicates the time (mm:ss) elapsed since the start of the video.
(4.9 MB AVI).

## Abbreviations

CIM: cell imagine medium
*KO*: knockout
MEF: mouse embryonic fibroblast
Syt I: synaptotagmin I
Syt VII: synaptotagmin VII
TIR-FM: total internal reflection fluorescence microscopy
*WT*: wild-type
